# Is Tranexemic Acid really helpful in major hemorrhage?

**DOI:** 10.12669/pjms.40.1.8888

**Published:** 2024

**Authors:** Faraz Mansoor

**Affiliations:** 1 Dr. Faaiz Ali Shah Email: faaizalishah@yahoo.com; 2 Naeemullah,; 3 Mian Amjad Ali,; 4 Mian Javed Iqba

We read with interest the study published in your esteemed journal about the Tranexamic acid.^1^ The authors highlighted the importance of recognizing the high risk surgical patients who are at risk of bleeding and they stressed the importance of using Tranexamic acid as part of the strategy to improve the outcome in this group of patients on a larger scale. In addition to this study, data from the recently published POISE-3 (Peri-Operative Ischemic Evaluation-3) trial also support the use of Tranexemic acid.^2^ However, we believe that this study has not added anything new in the literature and unless strong evidence is not available, Tanexamic acid should be used only in a clinical trial setting or as a part of the major haemmorhage protocol. We have the following comments and concerns about TXA.

There is no doubt that Tranexemic acid is widely used in clinical practice to reduce the risk of bleeding in high risk surgical patients. However, it is probably more important to ensure that the WHO surgical safety checklist is implemented at the national level, particularly at all the major tertiary centers of the country. In terms of the high risk surgical patients, we at Shaukat Khanum Memorial Cancer Hospital and Research center activate the major Haemorrhage protocol when there is more than 1.5 liters of blood loss and the patient is haemodynamically unstable.^3^ This MHP includes blood products such as packed red cells, platelets and fresh frozen plasma. In addition to this, Vit-K and Tranexamic acid is also administered. The aim is to keep the physiology in normal range while the source of the surgical bleeding is controlled. We believe that it would be practically impossible to study the outcome of Tranexamic acid alone in any setting as we cannot ignore the confounding factors in these complex clinical situations. Therefore, as a perioperative physician, I believe that implementation of WHO surgical safety checklist is more important and TXA can be added in this checklist in selected cases.

Secondly, the authors quite often forget to mention that the FDA only recommends TXA for heavy menstrual bleeding and as short-term prevention in patients with hemophilia. Moreover, there is insufficient data from the previous studies to support its widespread use. For example, there are selection bias issues in case of the famous CRASH-2 trial.^4^ This trial didn’t show any difference in the number of patients requiring blood transfusions or interventions between the two groups. So, if there was no change, how this agent could lead to a decrease in the mortality. While the CRASH-2 trial didn’t report any untoward effects of the TXA, it is surprising that they didn’t have any system to monitor these side effects as just 2% of the centers included in the trial had access to the rapid blood transfusion, damage control surgery, angiography interventions and critical care.^5^ It is also worth mentioning that those trials didn’t recommend the use of TXA because of its poor safety profile. For example, the HALT-IT trial which is the largest trial so far that recruited more than 12,000 patients with upper GI bleeding did not observe any benefit of using TXA between the two groups, and they actually observed that those patients who received TXA were more at risk of thromboembolic events and seizures.^6^

In summary, I believe that the TXA should be used when it is indicated and in case of its off-label use, clear guidelines should be developed at the organizational level in order to avoid any patient harm.

## REFERENCES

1. Shah FA, Naeemullah, Ali MA, Iqbal MJ. Efficacy of preoperative Tranexamic Acid in patients undergoing intertrochanteric hip fracture surgery: A randomized placebo controlled trial. Pak J Med Sci. 2023;39(6):1601-1605. doi: 10.12669/pjms.39.6.7667
2. Marcucci M, Painter TW, Conen D, Leslie K, Lomivorotov VV, Sessler D, et al. Rationale and design of the PeriOperative ISchemic Evaluation-3 (POISE-3): a randomized controlled trial evaluating tranexamic acid and a strategy to minimize hypotension in noncardiac surgery. Trials. 2022;23(1):101. doi: 10.1186/s13063-021-05992-1
3. Khan S, Allard S, Weaver A, Barber C, Davenport R, Brohi K. A major haemorrhage protocol improves the delivery of blood component therapy and reduces waste in trauma massive transfusion. Injury. 2013;44(5):587-592. doi: 10.1016/j.injury.2012.09.029
4. Collaborators CT, Shakur H, Roberts I, Bautista R, Caballero J, Coats T, et al. Effects of tranexamic acid on death, vascular occlusive events, and blood transfusion in trauma patients with significant haemorrhage (CRASH-2): a randomised, placebo-controlled trial. Lancet. 2010;376(9734):23-32. doi: 10.1016/S0140-6736(10)60835-5
5. Gruen RL, Jacobs IG, Reade MC; PATCH-Trauma study. Trauma and tranexamic acid. Med J Aust. 2013;199(5):310-311. doi: 10.5694/mja13.10747
6. Collaborators H-IT. Effects of a high-dose 24-h infusion of tranexamic acid on death and thromboembolic events in patients with acute gastrointestinal bleeding (HALT-IT): an international randomised, double-blind, placebo-controlled trial. Lancet. 2020;395(10241):1927-1936. doi: 10.1016/S0140-6736(20)30848-5

### Response from the authors:

We all the authors of article title, ”Efficacy of preoperative Tranexamic Acid in patients undergoing intertrochanteric hip fracture surgery: A Randomized Placebo Controlled Trial” published in Pakistan Journal of Medical Sciences volume 39, No.6 (1601-1605) are extremely thankful to the author for his critical review of our study. We would like to clarify the following points:

1. Our randomized trial added new knowledge to the literature as it was the first Primary WHO registered trial from low middle income country like Pakistan. Our sample population was relatively young when compared with other published RCTs where efficacy of Tranexamic Acid was assessed in elderly patients.^1^ Our sample size was adequate. We had perfectly mated study groups. We are hopeful that our results will be helpful in formulation of standard guidelines for routine use of Tranexamic Acid in selected patients in Dynamic Hip Screw (DHS) surgery in our country.
2. Our trial concluded that Tranexamic acid (TXA) significantly reduces the frequency of peri operative allogenic blood transfusion in patients undergoing DHS for intertrochanteric hip fractures. We believe that contemporary literature supports our results and provide sufficient evidence to advocate the use of Tranexamic Acid in selected patients with hip fracture surgery. Three recent systematic review and meta-analysis strongly support the use of Tranexamic Acid in hip fracture surgery as they concluded that Tranexamic Acid is effective in reducing peri operative allogenic blood transfusion rates and without any significant risk of mortality and thromboembolic complications.^2^-^4^
3. Although we were unable to measure the exact amount of blood loss in DHS surgery (limitation of our study) literature revealed that DHS surgery has been associated with perioperative blood loss of 1796.7 milliliter.^5^ This blood loss includes per operative visible blood loss and the hidden blood loss. Allogenic blood transfusion therefore is required in 20 to 60% of such patients.^1^,^6^ It is appropriate to mention that since most of the public sector hospitals in Pakistan lack the facilities to provide blood products such as packed red cells, platelets and fresh frozen plasma allogenic blood therefore is often transfused to stabilize the bleeding patients. Due to hazards of allogenic blood transfusions use of Tranexamic acid as an effective and safe alternative in selected patients is therefore justified.
4. We determined the efficacy of preoperative Tranexamic Acid in reducing perioperative allogenic blood transfusion frequency in patients with DHS surgery. The cofounders were controlled or excluded by randomization at the design level and by stratification at the analysis stage.
5. The CRASH-2 trial^7^ tested the efficacy and safety of Tranexamic Acid in trauma patients within eight hours of injury and two doses of Tranexamic Acid were administered. The primary outcome of Tranexamic CRASH-2 trial was mortality. In our trial we used only one dose of Tranexamic Acid pre operatively and none of our patient received Tranexamic Acid within eight hours of trauma. Furthermore, the primary objective of our study was to determine the efficacy of preoperative Tranexamic Acid in reducing preoperative allogenic blood transfusion frequency in patients with intertrochanteric fractures treated with DHS. Comparing CRASH-2 trial with our study therefore seems irrelevant.
6. The HALT-IT^8^ trial significantly differs from our trial. First, the target population was patients with acute gastrointestinal bleeding. Second, two doses of Tranexamic Acid were administered. Third, the primary outcome was five days mortality. Comparing the HALT-IT trial with our trial seems inappropriate.

We suggest that further local trials assessing the efficacy and safety of Tranexamic Acid should be conducted not only in Orthopaedic surgery but in other specialties too so that the indications of Tranexamic Acid are further expanded and verified.
